# Formulation and Evaluation of Novel Film Wound Dressing Based on Collagen/Microfibrillated Carboxymethylcellulose Blend

**DOI:** 10.3390/pharmaceutics14040782

**Published:** 2022-04-03

**Authors:** Kateřina Tenorová, Ruta Masteiková, Sylvie Pavloková, Klára Kostelanská, Jurga Bernatonienė, David Vetchý

**Affiliations:** 1 Department of Pharmaceutical Technology, Faculty of Pharmacy, Masaryk University, 61200 Brno, Czech Republic; masteikovar@pharm.muni.cz (R.M.); pavlokovas@pharm.muni.cz (S.P.); 507255@mail.muni.cz (K.K.); vetchyd@pharm.muni.cz (D.V.); 2 Department of Pharmaceutics, Faculty of Pharmacy, University of Veterinary and Pharmaceutical Sciences, 61200 Brno, Czech Republic; 3 Department of Drug Technology and Social Pharmacy, Faculty of Pharmacy, Lithuanian University of Health Sciences, 44307 Kaunas, Lithuania; jurga.bernatoniene@lsmuni.lt

**Keywords:** film wound dressing, collagen, microfibrillated carboxymethylcellulose, wound healing, blend films, solvent casting method

## Abstract

Collagen is essential as a physiological material in wound healing, so it is often used in wound management, mainly as a lyophilisate. Collagen also has excellent film-forming properties; unfortunately, however, its utilisation as a film wound dressing is limited because of its weak mechanical properties, especially in its wet state. For this reason, modifications or combinations with different materials are investigated. The combination of collagen with partially modified microfibrillar carboxymethylcellulose (CMC), which has not previously been described, provided a new possibility for strengthening collagen films and was the aim of this work. The collagen–CMC films based on three types of collagens, two plasticizers and two collagen. Plasticiser ratios were prepared using the solvent casting method; partially modified CMC served here as both a film-forming agent and a filler, without compromising the transparency of the films. The presence of microfibrils was confirmed microscopically by SEM. Organoleptic and physicochemical evaluation, especially in terms of practical application on wounds, demonstrated that all the samples had satisfactory properties for this purpose even after wetting. All the films retained acidic pH values even after 24 h, with a maximum of 6.27 ± 0.17, and showed a mild degree of swelling, with a maximum of about 6 after 24 h.

## 1. Introduction

Collagen is the most abundant protein in the human body [1,2]. It is the major structural component of the extracellular matrix (ECM), supporting connective tissues, such as tendons, ligaments, skin, and bones, and constituting approximately 30% of the body’s total protein [3]. Due to its natural origin and good chemical and physical properties, such as edibility, biodegradability, biocompatibility, low antigenicity, low irritation, and low cytotoxicity, collagen is widely used for different applications [4,5,6]. 

The principal function of collagen in the human body is to act as a scaffold in connective tissues; moreover, it is involved in physiological processes, such as wound healing and scar formation [7]. Collagen plays an essential role in all phases of the wound healing cascade because of its chemotactic function [8]. It stimulates cellular migration, which encourages debridement, angiogenesis, and re-epithelisation, and contributes to new tissue development [8,9]. Hence, collagen is a widely used material for preparing and producing modern wound dressings intended primarily for chronic wounds [10].

In chronic wounds, the healing is usually stalled at one of the healing stages, and the deposition of the newly formed collagen is delayed and prevented by many factors [7,8]. Recruitment of fibroblasts is retarded; moreover, the expression of the collagen gene in fibroblasts is suppressed. In addition, elevated levels of matrix metalloproteinases (MMPs) usually occur [10,11]. Although these proteases are essential for routine wound healing and new tissue formation because of their essential function in breaking down the damaged ECM, elevated levels of MMPs contribute to the destruction of a healthy and newly formed ECM. In conclusion, elevated MMP levels are associated with delayed wound healing and increased wound size [8,9]. Collagen dressings provide an alternative source of collagen to the wound, which the high levels of MMPs can degrade, so the endogenous collagen is not destroyed and the wound continues in the normal healing process [7,8,12]. Collagen dressings are available in various forms, including sponges, sheets, powders, hydrogels, etc., and collagen is also often combined with other substances [8,13]. 

Films are moisture vapor- and oxygen-permeable wound dressings, which are used especially for minor burns and superficial wounds with no or a small amount of exudate. They are impermeable to microorganisms and liquids, and their transparency and flexibility allow easy assessment of the wound. They can also be used in surgery to cover sutures or protect the skin, including from shearing or covering intravenous catheters [14]. Currently, film wound dressings are mainly manufactured from synthetic polyurethane, but there are increasing efforts to prepare films from materials of natural origin [15,16]. 

Collagen has excellent film-forming properties, which is why collagen films are extensively used in tissue engineering and as edible coatings in the food industry [6,17]. However, the physicochemical properties collagen films present some disadvantages, limiting the applications of collagen in wound dressings [6]. Especially after contact with exudate, poor handling properties of collagen films can be a problem [18]. For this reason, suitable physical or chemical modifications are needed to improve collagen’s properties. Crosslinking or combining with supporting materials would solve the problem. Crosslinking has been used to modify the molecular structure of collagen, minimise degradation, and enhance mechanical stability. There are several options, such as enzymatic, physical, or chemical crosslinking, but in the case of chemical crosslinking, crosslinking agents may not be physiologically beneficial [19,20]. Potential combinations with different support materials have also been investigated. For example, Wang et al. proved that hydroxyapatite addition significantly improved the tensile strength of the composite collagen films [21]. 

The most promising possibility is the combination with carboxymethylcellulose. Carboxymethylcellulose (CMC) is a cellulose derivative that is widely applied in food, cosmetics, medicine, pharmaceuticals, and tissue engineering [22]. CMC is generally regarded as a non-toxic, non-irritant, biocompatible, and biodegradable material with a low cost compared to other natural polymers, and it has a good film-forming capacity. These properties make CMC a suitable material for a new wound dressing development [22,23,24]. In our previous studies, bilayer textile carboxymethylcellulose–collagen films and collagen films with the acidic form of carboxymethylcellulose in the form of a non-woven textile, which have satisfactory properties for application as wound dressings, have been prepared [25,26]. Our research team also used partially substituted NaCMC in the form of a non-woven textile (Hcel^®^ NaT), which led to the strengthening and improvement of the mechanical properties of the resulting films, due to the microfibrillar structure of CMC. Moreover, additional acidification of dispersion enhanced handling properties even more, as it caused the formation of an insoluble matrix of the acidic form of carboxymethylcellulose (HCMC) [16,23]. As it was confirmed in some studies [27] that the partial insolubility and fibrous structure of supporting materials should lead to films with improved mechanical properties, including collagen films [28], our assumption was that combination of collagen with an HCMC matrix could enhance the handling properties of the collagen-based film wound dressing.

All the aforementioned facts and assumptions led to the objective of the presented research, which was to prepare a novel film wound dressing based on a collagen and microfibrillated carboxymethylcellulose blend as a modern wound dressing. To our knowledge, such a combination of collagen and microfibrillated CMC (we use the general term carboxymethylcellulose because both forms of NaCMC and HCMC are represented in our films) in the form of a non-woven textile has not been investigated yet. The physicochemical properties of the films were evaluated, especially in terms of the application of the film to the wound in the wet state. The influence of three different types of collagens and two plasticisers, as well as their ratio, on the film properties and quality attributes was evaluated. 

## 2. Materials and Methods

### 2.1. Composite Film Preparation

Porcine, bovine, and equine collagen in the form of a gel was supplied by Collado spol. s.r.o. (Brno, Czech Republic). The partially substituted (DS 0.4–0.5) sodium carboxymethylcellulose (NaCMC) in the form of a non-woven textile (Hcel^®^ NaT) was obtained from Holzbecher, spol. s.r.o. (Czech Republic). Glycerine, macrogol 300, ethanol 96%, and hydrochloric acid (all Ph. Eur. grade) were purchased by Fagron, a.s. (Olomouc, Czech Republic). All other chemicals used in the experiment were of analytical grade, according to *European Pharmacopoeia*. 

The composite films based on polymers of natural origin—collagen and carboxymethylcellulose—were prepared using the solvent casting method. The process consists of two main steps: the preparation of CMC dispersion, followed by the preparation of a final dispersion for casting. This final dispersion contains the combination of CMC dispersion and collagen gel, whose concentration was determined in advance. First, the polymer dispersion of NaCMC was made. It consisted of 1% *w*/*w* CMC and 1% *w*/*w* plasticiser (glycerine or macrogol 300) in purified water. The sodium carboxymethylcellulose, in the form of a non-woven textile, was cut into small parts and a plasticiser solution in water was poured over it (80 °C). This dispersion was heated in a water bath for 3 h to maintain a temperature of 80 °C, and was left to swell at an ambient temperature for 24 h. The next day, the NaCMC dispersion was homogenised by a Cito-UNGUATOR 2000 (SMS Heiztechnik GmbH, Zella-Mehlis, Deutschland) set on the “Gel” program. Then, acidification of the dispersion to pH 3 was carried out, using a 5% and 1% solution of hydrochloric acid (HCl); after that, the dispersion was homogenised again. The dispersion prepared in this way was then combined with a collagen gel as follows: the calculated amount of collagen gel was added to the dispersion of CMC (collagen–CMC ratio 1:1), together with an additional amount of plasticiser (macrogol 300 or glycerine), to reach the required ratio to collagen. After homogenisation, the dispersion was cast on a rectangular plastic Petri dish or plastic tray to maintain a concentration of collagen 0.003 g/cm^2^. The solvent was left to evaporate at ambient conditions (room temperature) for approximately 48 h. The dried films were peeled from the Petri dishes or trays, and the samples of required dimensions suitable for each evaluation test were punched using steel punches. The films were stored in closed bags and boxes to await testing. The composition of prepared films is summarised in Table 1.

### 2.2. Organoleptic and Microscopic Evaluation

First, all prepared films were assessed by the visual examination in the dry and wet states. The structures and film surface properties were characterised by SEM. The samples were placed on aluminium stubs with double-sided adhesive carbon tape and observed using a scanning electron microscope (MIRA3, Tescan Brno, s.r.o., Czech Republic). Obtained signals of the samples were produced by secondary electrons (SE) at 3 kV voltage and 100×, 500×, and 1000× magnifications.

### 2.3. Film Thickness

The thickness was assessed using an Elcometer 456 coating thickness gauge (Elcometer Limited, Manchester, UK). The sample was laid on a solid base and fitted with the probe of the thickness gauge. The thickness was measured both in dry and wet conditions (intervals of 15 min, 1 h, 3 h, 8 h, and 24 h). The measurements were made at 30 locations over the area of the sample. The results were used for recalculation data obtained from the texture analysis to value 100 μm. 

### 2.4. Film Weight and Uniformity of Mass

The uniformity of mass evaluation for film wound dressings is not defined in *European Pharmacopoeia*. For this reason, the evaluation of the films was done according to the adapted test (2.9.5. Uniformity of mass of single-dose preparations) described in *European Pharmacopoeia*. Twenty pieces, each 2.5 × 2.5 cm in size, were rigorously cut from random locations on the prepared films. Each sample was weighed on an analytical scale (KERN 870–13, Gottl. KERN & Sohn GmbH, Germany) with an accuracy of 4 decimal places, and the average mass ± SD was assessed. The percentage of deviation from the average mass was calculated. Limits for film-coated and uncoated tablets with an average mass between 80 and 250 mg were applied (even for samples with a weight under 80 mg). According to *European Pharmacopoeia*, no more than 2 of the individual masses could deviate from the mean by more than 7.5%, and none by more than twice that percentage [29]. Results are presented as mean values, with the minimum and maximum values expressed in mg and % of film weight.

### 2.5. Surface pH

The surface pH of the prepared films was measured using a contact pH meter (Flatrode, Hamilton, CH) after wetting with a drop of purified water. This measurement was conducted three times for each sample, and the results are presented as average values and SDs. 

The measurement of surface pH alteration in determined time intervals was performed using an artificial wound model developed by Vinklárková et al. [16]. The model aimed to simulate real wound environment conditions. It was based on a Petri dish with a sponge soaked in a test medium-buffered salt solution with a pH of 7.2 (BSS), according to *European Pharmacopoeia*. The sponge’s surface was rough, in order to simulate the surface of the wound, and the sponge was soaked with 20 mL of BSS, imitating the wound exudate. Film squares measuring 2.5 × 2.5 cm were cut and put on the wound model. The lid was used to cover the Petri dish to prevent the evaporation of the liquid. At determined time intervals (15 min, 1, 3, 5, 8 h), surface pH was measured three times. The results are presented as average values and SDs of each sample.

### 2.6. Swelling Properties of Films

The measurement of swelling properties was carried out using the artificial wound model described above (Petri dish, sponge soaked with a 20 mL BSS). Cut samples of the films, with dimensions of 6.25 cm^2^ (2.5 × 2.5 cm), were weighed on an analytical scale (KERN 870–13, Gottl. KERN & Sohn GmbH, Balingen, Germany) in dry conditions (Wd). After that, the samples were placed in the artificial wound model. At certain time intervals (15 min, 1, 3, 5, 8 h), swollen films were weighed (Ws). The degree of swelling (Sw) of the film was calculated using the following equation:

Sw = (Ws − Wd)/Wd.(1)

Swelling experiments were conducted in triplicate and average results were reported.

### 2.7. Mechanical Properties

To evaluate the mechanical properties of the films, texture analysis was used. Both measurements, tensile testing and puncture testing, were made in dry and wet states (3 h of swelling on an artificial wound model). A CT 3 Texture Analyzer (Brookfield, Middleboro, MA, USA) equipped with TexturePro CT software and a 4.5 kg load cell was used for tensile testing. Film samples (40 × 10 mm) were mounted between the upper and lower grips of the TA-DGA probe, which was positioned at 2 cm. The lower grip was held stationary, and the film samples were stretched by the upper grip, which moved at a speed of 0.5 mm/s, to pull apart the strip of the film until it broke. The force and work done during this process and the deformation (elongation) at the moment of tearing were measured five times for each sample. The results are presented as mean values and SDs for each parameter of the film, recalculated for a uniform thickness of 100 μm.

For the puncture test, a CT 3 Texture Analyzer with a TA39 cylindrical probe (2 mm diameter, probe motion speed 0.5 mm/s) was used. The films were cut into squares measuring 25 × 25 mm, and fixed in the JIG TA-CJ holder. The force needed to puncture the film, the work done during this process, and the deformation at the moment of penetration were measured and repeated five times for each sample. The results are presented as mean values and SDs for each parameter of the film, recalculated for a uniform thickness of 100 μm.

### 2.8. Data Analysis

The aim of data processing was to determine the influence of formulation parameters on the selected film physicochemical properties by means of univariate and multivariate statistical techniques.

The effects of the collagen type and plasticiser type, as well as their ratio, were verified using the analysis of variance (ANOVA) or *t*-test. The ANOVA method was used to test the main trend in the whole data set, which was subsequently supplemented by testing individual sample pairs using a *t*-test. The *p*-values as the outputs of statistical testing are given in parentheses for each discussed film attribute throughout the Results and Discussion section. The significance level was set to the value of α = 0.05, so a *p*-value lower than α indicates the statistical significance of the tested parameter.

To assess the mutual effects between the selected input and output variables, principal component analysis (PCA) was carried out on data after standardisation. The PCA method was supported by visualisation, using a loadings plot and a scores plot, on the basis of which the correlation data structure could be assessed. The similarities between samples’ properties can be examined based on the distribution of corresponding points in the scores plot. Scores spaced close to each other indicate the high degree of samples’ similarity, while samples with scores far apart are rather different. The angle between two vectors in the loadings plot can be interpreted as follows: the lower the value of the angle between arrows, the higher the positive correlation between samples’ characteristics. The right angle between vectors suggests no correlation. An angle close to 180° confirms a negative correlation. In terms of vector length, a higher value indicates a higher proportion of explained variability. The data analysis was performed utilising software R, version 4.0.4 [30]. 

## 3. Results and Discussion

### 3.1. Organoleptic and Microscopic Evaluation

**Figure 1:**
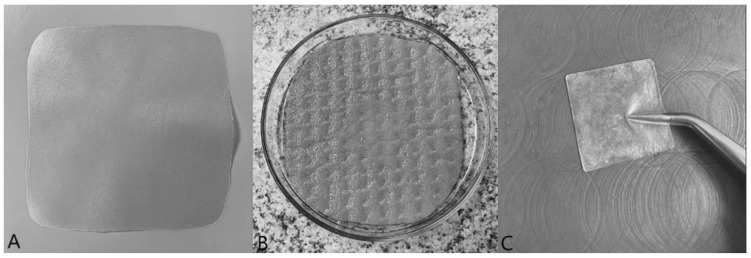
(**A**) The appearance of the prepared film (8 × 8 cm). (**B**) Swelled film after application on artificial wound model (2.5 × 2.5 cm). (**C**) Appearance and cohesiveness of a film (2.5 × 2.5 cm) after wetting.

An ideal film dressing must be homogenous, with a smooth surface, and should be similar to the skin. Other important properties of film wound dressings are transparency, flexibility, and cohesiveness, especially when wetted [14,31]. Film transparency allows easy observation and assessment of wounds without replacement, and flexibility is essential for good manipulation and adaptation to the wound roughness [32,33]. However, film wound dressings could have other properties, including antibacterial, antioxidant, and anti-inflammatory effects, etc. [34,35]. The visual examination did not demonstrate any significant differences between the prepared films. All of them were homogenous, flexible, and translucent, with smooth surfaces. Dry films were firm, resilient, and pleasant to the touch (Figure 1A). The difference in the appearance of the films did not differ within individual collagens, but films with macrogol gave a firmer and coarser impression in comparison to films with glycerine. However, films with glycerine were more flexible than macrogol ones. All the films become soft and pliable after wetting (Figure 1B). They had good adherence to the skin, had good manipulation properties, and were coherent even after 24 h on an artificial wound model (Figure 1C). The differences between the individual types of collagen or plasticisers and their ratios were not noticeable.

**Figure 2:**
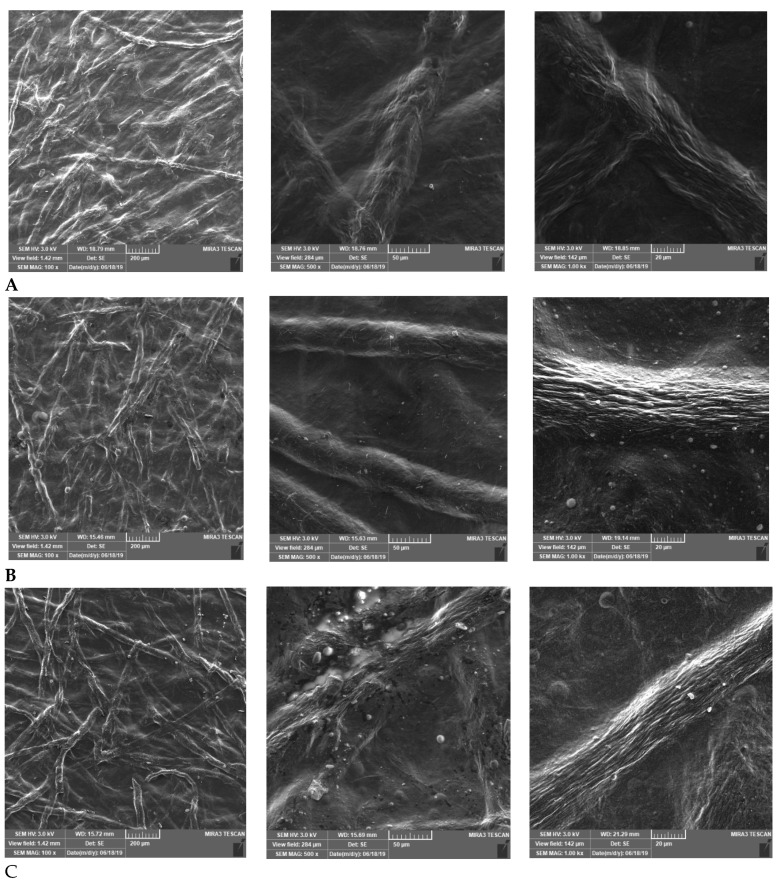
Microscopic appearance of the films: (**A**) bovine collagen; (**B**) porcine collagen; and (**C**) equine collagen (all magnifications 100×, 500×, and 1000×, from left to right).

Observation of the microscopic appearance of prepared films by SEM confirmed that the partially substituted CMC maintained a fibrous nature. Digital images showed well visible microfibrous structures in all samples (Figure 2). Our assumption has therefore been confirmed. Microfibrous CMC leads to the strengthening and improvement of the mechanical properties of the resulting film wound dressing [16,23].

### 3.2. Film Thickness

Uniform thickness means a correct preparation method, so it is an essential parameter from the technological perspective [36,37]. The thicknesses of all prepared films in the dry state ranged from 60.8 ± 6.6 µm to 120.2 ± 11.2 µm (Table 2). Thickness was significantly dependent on the type of the plasticiser and its ratio, as well as the type of collagen (*p* < 0.001 in all cases). All the samples with ratios of 1:3 were thicker than samples with ratios of 1:2, and samples with macrogol 300 were thicker than the films with glycerine. Films from porcine and bovine collagens were thicker than the equine ones. The thickness measurement resulted in a dry state, as evidenced by low SD values, which showed the sufficient reproducibility of the film preparation method. The thicknesses of all prepared films after wetting were measured at certain time intervals (15 min, 1, 3, 8, 24 h). High SD values characterised these results, due to partly uneven swelling and more difficult measurement conditions. The thicknesses of all samples increased over time and were dependent on the collagen type (bovine and porcine samples were thicker than equine ones; *p* < 0.001 at all time points), as well as the ratio of the plasticiser (the samples with ratios of 1:3 were generally thicker than samples with ratios of 1:2; *p* < 0.05 in most cases). However, the effect of the type of plasticiser on the wet thickness was not confirmed as statistically significant by ANOVA or *t*-tests (*p* > 0.05 in most cases, especially when measuring at higher time intervals).

### 3.3. Film Weight and Uniformity of Mass

The weight of prepared film samples (25 × 25 mm) ranged from 66.3 ± 3.3 mg to 98.1 ± 4.3 mg, with an uptrend in plasticiser concentration (ratio) increase (Table 3). All samples (ratio 1:3) weighed more than samples with ratios of 1:2 (*p* < 0.001 for each comparison). In terms of collagen type effect, it was evaluated by ANOVA as statistically significant (*p* < 0.001), but an ambiguous trend was revealed, as a significant interaction between collagen type and the collagen–plasticiser ratio was present. The type of plasticiser used did not significantly affect the weights of the samples when the summary was tested using ANOVA (*p* = 0.650).

Deviations from the mean weight for each sample were calculated for the uniformity of the mass test. Table 3 shows the minimum and maximum deviation from the mean weight of samples, expressed in mg and %. All the films met the test requirements [29].

### 3.4. Surface pH

Evaluation of pH is an essential test because it shows the real effect of the dressing on the wound. In this case, alteration to the film’s surface pH is especially important because it shows us the ability of the wound dressing to maintain pH in the acidic range even for a more extended period. It is generally known that pH value affects the process of wound recovery. The pH value of the wound significantly affects a matrix’s metalloproteinase (MMP) activity, as well as its tissue inhibitors and fibroblast activity. It also affects keratinocyte proliferation, microbial proliferation, and immunological responses in a wound [38,39]. An alkaline wound environment, tissue destruction, and increased risk of infection are commonly associated with chronic wounds. It is generally proven that the acidic environment helps in wound healing by preventing infection and decreasing the level of MMPs [40,41]. We used the buffered salt solution with a pH of 7.2 because it is similar to wound fluid (exudate) in terms of pH value and ion content. The aim was to test the films’ ability to maintain acidic pH values even after 24 h in buffering conditions. Values of surface pH after the wetting of all prepared films are shown in Table 4. The pH values ranged from 2.10 ± 0.07 (Sample E-M-2) to 3.22 ± 0.01 (Sample P-G-3), so the films’ surfaces were acidic. A statistically insignificant influence of plasticiser type on the collagen–plasticiser ratio was found (*p* > 0.05 in almost all cases). On the other hand, the effect of collagen type on pH value was assessed as significant, with pH increases in the following order: equine < bovine < porcine collagen. Alterations to the surface pH of the films during determined time intervals, in conditions simulating wound environments, are shown in Table 4. The table demonstrates that, although the pH increased within the time intervals, all the films retained acidic pH values even after 24 h, achieving pH values of 4.24 ± 0.13 (B-M-2) and 6.27 ± 0.17 (E-G-3), respectively. This indicates the ability of the films to keep their pH in the acidic range, so we can consider them as pH-modulating wound dressings. However, equine collagen films could maintain pH in the acidic range less successfully than the bovine and porcine ones (*p* < 0.001). The effects of plasticiser type and collagen–plasticiser ratio on the pH remained insignificant over time.

### 3.5. Swelling Properties of Films

**Figure 3:**
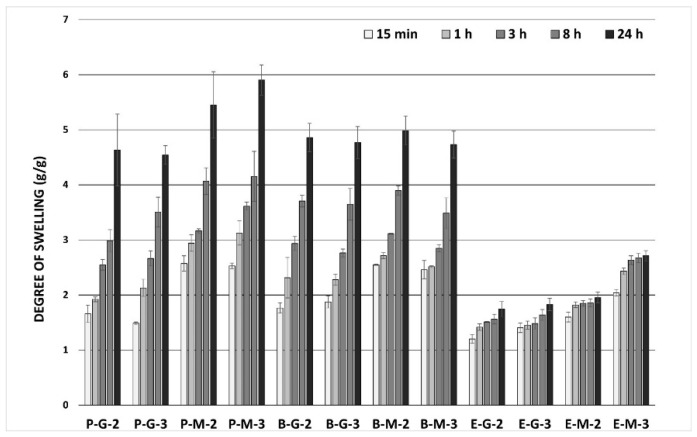
Swelling behaviour of the prepared films.

An essential property of a wound dressing is its swelling behaviour, which indicates the ability to absorb exudate and provide a moist environment in the wound. The liquid uptake of the film prevents drying out and creates optimal conditions for moist wound healing [42,43]. In general, high absorption values are not expected from film wound dressings, but adequate efficiency in this area is also desirable [14]. The degree of swelling (Sw) was evaluated in a buffered salt solution with pH of 7.2 because this is similar to wound fluid (exudate) in terms of pH value and ion content. Determined swelling values of the samples should reliably reflect those in real wounds [44]. Films from porcine and bovine collagens exhibited a mild degree of swelling, maintaining their structural integrity for the required period. This indicates moderate holding capacity for the liquid (Figure 3). Compared to porcine and bovine films, films from equine collagen exhibited a lower degree of swelling. Swelling values of all films from porcine and bovine collagens increased gradually up to 24 h. In the case of equine collagen, the swelling degree increased only slightly with time. In general, samples from equine collagen were less absorbent and showed lower swelling values (*p* < 0.001 at all time points in general, as well as *p* < 0.05 for each pair of samples compared). Samples with macrogol (P-M-2, P-M-3, B-M-2, B-M-3, E-M-2, E-M-3) showed a slightly higher swelling than the glycerine ones (*p* < 0.001 at all time points). The effect of the plasticiser ratio on the swelling properties was negligible (*p* > 0.05 at all time points).

### 3.6. Mechanical Properties

**Figure 4:**
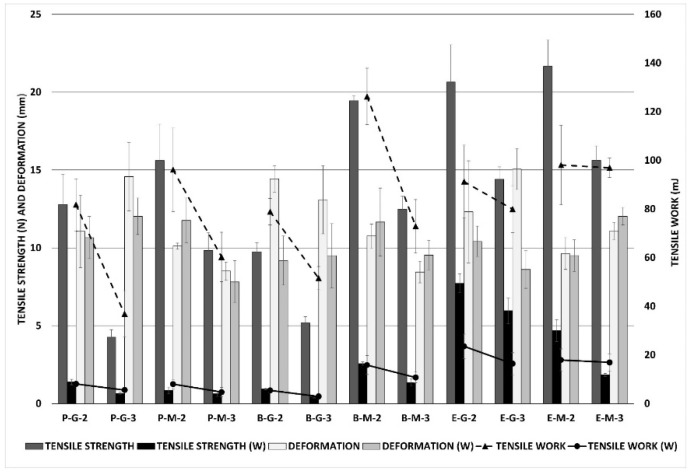
Mechanical properties of films as observed through tensile testing. Comparison of tested parameters (strength, work, deformation) in dry and wet states (W).

Characterisation of prepared films’ mechanical properties is an important evaluation for practical application to wounds. Film dressings are required to be durable, soft, flexible, pliable, stress-resistant, and elastic, due to adaptation to different parts of the body [45,46,47]. Among all the characteristics, the deformation values are the most important. The higher the deformation values, the more flexible and more adaptable the film is. Mechanical qualities are essential, not only in a dry state, but also after wetting (contact with wound exudate). The film should remain cohesive even after wetting, to ensure its easy removal from the wound [48,49]. The mechanical properties of the samples were assessed using two different approaches: tensile testing (tensile work, tensile strength, and elongation), and puncture testing (puncture work, puncture force, and deformation); and in two different conditions: in dry state and wet state (after three hours on the artificial wound model). All values were then recalculated, for a uniform thickness of 100 μm. The tensile testing results from the dry and wet states are shown in Figure 4, which demonstrates the mechanical properties of the films. In most cases, equine samples reached higher values of tensile strength and tensile work than the bovine and porcine ones (*p* < 0.001 in both cases), but the differences between the values of elongation (deformation) were not statistically significant (*p* = 0.277) when testing via ANOVA. The influence of the plasticiser and its ratio depends on the evaluation of a specific parameter. Films with macrogol 300, as well as films with a plasticiser ratio of 1:3, generally had higher values of tensile strength and tensile work than the others (*p* < 0.001 for all comparisons). In contrast, the effect of plasticiser type was the opposite: higher deformation for films with glycerine than the films with macrogol (*p* < 0.001), while the influence of the collagen–plasticiser ratio on the deformation (elongation) values was negligible (*p* = 0.334). Not surprisingly, lower tensile strength values and less work were observed in all wetted films (W). Deformation (elongation) and its changes after wetting were negligible. It was evident that wetted equine films reached higher values of tensile strength and tensile work than wetted films from porcine and bovine collagens (*p* < 0.001 in all cases). The effect of the collagen–plasticiser ratio on film deformation was statically insignificant; nevertheless, higher values of tensile work and strength were confirmed for ratios of 1:2 than for ratios of 1:3. The effect of plasticiser on the properties of the films in the wet state was not proven (*p* = 0.362 for deformation; *p* = 0.125 for work; *p* < 0.001 for strength, but the trend for this variable is not clear).

**Figure 5:**
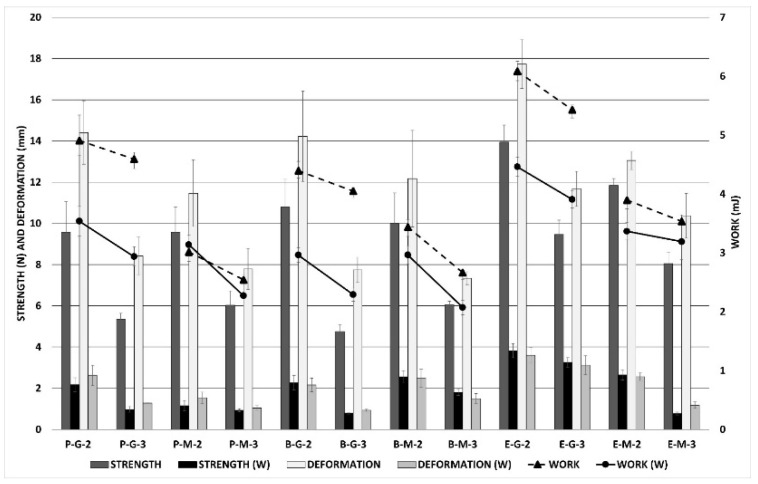
Mechanical properties of films as observed through puncture testing. Comparison of tested parameters (strength, work, deformation) in dry and wet states (W).

Figure 5 demonstrates the mechanical properties of the films after puncture testing in dry and wet states. Films prepared from equine collagen had slightly better mechanical properties than bovine and porcine ones (*p* < 0.001 in almost all cases). Regarding puncture work and force, the influence of the type of the plasticiser was negligible (*p* > 0.05 in most cases). In contrast, the plasticiser type had a statistically significant impact on deformation value (*p* < 0.05 for all comparisons); specifically, the films with glycerine showed more significant deformation than samples containing macrogol. The greatest statistically significant differences were noticeable between films with different plasticiser ratios; ratios of 1:2 had better mechanical properties than ratios of 1:3 (all *p*-values lower than 0.05). In addition to tensile testing, lower values of all the tested parameters were observed in the wet state (W). The influences of the collagen type and the plasticiser ratio were also confirmed (*p* < 0.05 for almost all compared groups). However, the influence of the plasticiser type on puncture work and force was inconclusive (*p* < 0.05 in some cases, but no clear trend can be observed in data), while the significant effect of this variable on the deformation value was confirmed (*p* < 0.001 in a majority of cases).

### 3.7. Principal Component Analysis

**Figure 6:**
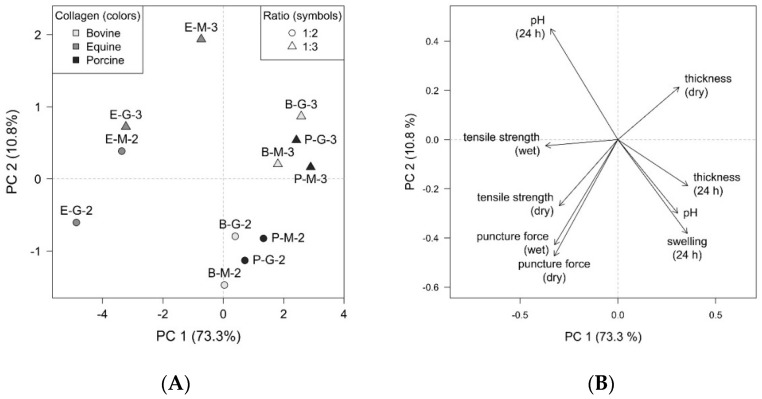
(**A**) PCA scores plot—collagen type differentiated by colours, collagen–plasticiser ratio differentiated by symbols; and (**B**) PCA loadings plot—selected properties of the films.

The multivariate analysis also confirmed the conclusions mentioned above. For a better view of the interdependencies and mutual relationships between the formulation parameters and the properties of the individual samples, the PCA method was employed. Graphical representation is provided by a PCA scores plot (Figure 6A), which displays the layout of objects, and a PCA loadings plot (Figure 6B), which shows the correlation structure of variables. In the resulting model, the first two principal components (PC) comprise 84.1 % of total variability, which can be considered sufficient to provide a reliable data interpretation.

As shown in the graphs, the two main correlation trends in the data are clear. In the direction from the upper left quadrant to the lower right quadrant (Figure 6A), the effect of collagen type is dominant. The vectors of pH, pH (24 h), swelling (24 h), and thickness (24 h) correspond to this direction (Figure 6B). In the approximately perpendicular direction, i.e., from the upper right quadrant to the lower left quadrant (Figure 6A), the collagen–plasticiser ratios of 1:2 and 1:3 can be distinguished. This direction in the loadings plot (Figure 6B) is represented by thickness (dry), tensile strength (dry), and puncture force (dry/wet) arrows.

The grouping of objects depending on collagen type showed that samples with equine (E) collagen are considerably different from samples containing porcine (P) and bovine (B) collagens. At the same time, the latter two are more similar. The pH tested in 24 h was higher for E samples, while the surface pH after wetting, the swelling degree, and the thickness measured in 24 h were higher in B and P samples. In terms of the collagen–plasticiser ratio, the higher values of tensile strength in a dry state and puncture force in dry and wet states for the ratio of 1:2 were observed. In comparison, the thickness in a dry state showed higher values for samples with ratios of 1:3. The effect of the collagen type combined with the distinction between ratios of 1:2 and 1:3 was manifested primarily in the tensile strength in a wet state. Other influences were evaluated as relatively minor based on the given PCA output.

## 4. Conclusions

This study aimed to prepare and evaluate a novel film wound dressing based on a collagen and microfibrillated carboxymethylcellulose blend. The films were successfully prepared by the solvent casting method, with three different types of collagen and two plasticisers. All the samples had good organoleptic properties and satisfactory mass content uniformity. The microscopic evaluation confirmed the presence of the microfibrillar structure of CMC, which ultimately led to sufficient flexibility and cohesiveness after wetting, which is necessary for wound application. All the films maintained an acidic pH and exhibited a low degree of swelling, which is typical for this type of wound dressing. All the samples demonstrated good mechanical durability even after wetting, which is essential for practical application to wounds. Based on the statistical testing and multivariate analysis, it can be concluded that the observed properties are primarily influenced by the collagen type (considerable difference between equine collagen and the other two types) and, to a lesser extent, by the collagen–plasticiser ratio. Based on this experiment’s positive and promising results, further research should continue to evaluate stability after gamma radiation, physical sterilisation, and long-term stability testing during storage at different temperatures and humidity conditions.

## Figures and Tables

**Table 1:** Composition of 100 g casting dispersions used for film wound dressing preparation.

Sample	Collagen Source	Amount of Collagen (g)	Amount of CMC (g)	Plasticiser	Amount of Plasticiser (g) (CMC Dispersion + Added Extra)	Collagen–Plasticiser Ratio
P-G-2	porcine	1.0	1.0	glycerine	1.0 + 1.0	1:2
P-G-3	porcine	1.0	1.0	glycerine	1.0 + 2.0	1:3
P-M-2	porcine	1.0	1.0	macrogol 300	1.0 + 1.0	1:2
P-M-3	porcine	1.0	1.0	macrogol 300	1.0 + 2.0	1:3
B-G-2	bovine	1.0	1.0	glycerine	1.0 + 1.0	1:2
B-G-3	bovine	1.0	1.0	glycerine	1.0 + 2.0	1:3
B-M-2	bovine	1.0	1.0	macrogol 300	1.0 + 1.0	1:2
B-M-3	bovine	1.0	1.0	macrogol 300	1.0 + 2.0	1:3
E-G-2	equine	1.0	1.0	glycerine	1.0 + 1.0	1:2
E-G-3	equine	1.0	1.0	glycerine	1.0 + 2.0	1:3
E-M-2	equine	1.0	1.0	macrogol 300	1.0 + 1.0	1:2
E-M-3	equine	1.0	1.0	macrogol 300	1.0 + 2.0	1:3

**Table 2:** Film thickness.

Sample	Thickness (µm)					
	Dry State	15 min	1 h	3 h	8 h	24 h
P-G-2	84.7 ± 8.3	146.8 ± 22.1	148.7 ± 22.4	199.7 ± 18.8	231.2 ±27.7	331.5 ± 66.5
P-G-3	91.6 ± 8.6	153.0 ± 19.3	171.0 ± 16.5	233.7 ± 23.3	247.8 ± 31.3	382.5 ± 61.9
P-M-2	97.4 ± 7.9	185.5 ± 12.3	191.2 ± 23.8	192.3 ± 20.0	280.9 ± 36.3	332.0 ± 47.0
P-M-3	120.2 ± 11.2	257.1 ± 37.8	272.8 ± 29.0	278.1 ± 20.4	311.6 ± 71.7	386.5 ± 108.3
B-G-2	85.1 ± 9.2	229.6 ± 38.5	233.3 ± 33.3	262.8 ± 26.5	291.8 ± 31.9	362.3 ± 76.7
B-G-3	96.7 ± 7.6	199.1 ± 35.6	249.6 ± 32.5	284.8 ± 35.1	300.0 ± 47.6	468.3 ± 72.3
B-M-2	91.1 ± 6.6	234.6 ± 15.6	236.4 ± 39.3	250.3 ± 18.9	254.5 ± 20.9	402.2 ± 61.9
B-M-3	117.2 ± 10.1	279.7 ± 70.1	299.1 ± 37.4	307.3 ± 28.0	332.2 ± 28.3	421.1 ± 76.7
E-G-2	60.8 ± 6.6	98.6 ± 18.0	106.2 ± 14.7	124.7 ± 8.6	135.3 ± 13.8	135.6 ± 14.8
E-G-3	69.5 ± 7.6	154.2 ± 16.7	161.7 ± 16.8	178.0 ± 16.6	179.1 ± 17.6	191.2 ± 27.1
E-M-2	82.3 ± 6.9	118.4 ± 11.6	121.6 ± 10.7	127.6 ± 10.6	136.3 ± 16.1	139.9 ± 19.5
E-M-3	100.2 ± 9.4	154.7 ± 22.3	175.1 ± 26.1	194.9 ± 14.8	211.9 ± 22.5	220.7 ± 19.2

**Table 3:** Uniformity of mass.

Sample	Average Weight (mg)	Min. Weight		Max. Weight		Compliance with *European Pharmacopoeia* Limit
		mg	% ^a^	mg	% ^a^	
P-G-2	71.4 ± 2.0	67.5	−5.4	73.9	+3.5	Yes
P-G-3	98.1 ± 4.3	91.4	−6.8	106.1	+8.1 ^b^	Yes
P-M-2	79.5 ± 3.3	75.6	−4.9	86.6	+8.9 ^b^	Yes
P-M-3	93.6 ± 1.9	89.5	−4.4	96.8	+3.4	Yes
B-G-2	83.2 ± 4.1	77.5	−6.8	89.6	+7.7 ^b^	Yes
B-G-3	95.2 ± 5.3	89.4	−6.1	107.1	+12.5 ^b^	Yes
B-M-2	70.4 ± 3.5	65.2	−7.4	76.4	+8.5 ^b^	Yes
B-M-3	94.6 ± 5.7	82.7	−12.6^b^	101.6	+7.3	Yes
E-G-2	66.3 ± 3.3	61.4	−7.5	73.0	+10.0 ^b^	Yes
E-G-3	91.7 ± 3.6	87.2	−4.9	98.8	+7.7 ^b^	Yes
E-M-2	75.2 ± 4.1	68.3	−9.1^b^	80.3	+6.8	Yes
E-M-3	94.5 ± 2.3	89.6	−5.2	98.8	+4.5	Yes

^a^ Deviation from the average; ^b^ Maximum two samples were out of the limit ±7.5%.

**Table 4:** pH after wetting and pH alterations in determined time intervals.

Sample	pH					
	After Wetting	pH Alterations in Determined Time Intervals				
		15 min	1 h	3 h	8 h	24 h
P-G-2	3.15 ± 0.02	3.73 ± 0.01	3.81 ± 0.03	3.78 ± 0.03	3.82 ± 0.04	4.46 ± 0.24
P-G-3	3.22 ± 0.01	3.72 ± 0.05	3.86 ± 0.06	3.71 ± 0.09	4.27 ±0.07	4.58 ± 0.18
P-M-2	3.05 ± 0.03	3.60 ± 0.11	3.78 ± 0.03	3.90 ± 0.06	3.83 ± 0.08	4.44 ± 0.12
P-M-3	3.04 ± 0.04	3.55 ± 0.04	3.76 ± 0.03	3.99 ± 0.06	3.78 ± 0.08	4.31 ± 0.07
B-G-2	2.45 ± 0.08	3.60 ± 0.03	3.49 ± 0.19	3.64 ± 0.04	3.87 ± 0.06	4.51 ± 0.17
B-G-3	2.62 ± 0.02	3.73 ± 0.17	3.46 ± 0.11	3.69 ± 0.18	3.80 ± 0.06	4.39 ± 0.08
B-M-2	2.50 ± 0.03	3.54 ± 0.07	3.38 ± 0.01	3.67 ± 0.06	3.65 ± 0.03	4.24 ± 0.13
B-M-3	2.63 ± 0.06	3.32 ± 0.03	3.36 ± 0.04	3.46 ± 0.06	3.75 ± 0.02	4.38 ± 0.18
E-G-2	2.18 ± 0.05	4.05 ± 0.03	4.07 ± 0.08	4.63 ± 0.19	5.94 ± 0.11	6.21 ± 0.17
E-G-3	2.19 ± 0.11	3.96 ± 0.14	4.01 ± 0.05	4.23 ±0.04	5.93 ± 0.18	6.27 ± 0.17
E-M-2	2.10 ± 0.07	3.80 ± 0.24	4.14 ± 0.06	4.24 ± 0.05	4.61± 0.36	6.02 ± 0.15
E-M-3	2.20 ± 0.07	3.75 ± 0.07	3.93 ± 0.04	3.97 ± 0.24	4.11± 0.27	5.99 ± 0.05

## Data Availability

Data are contained within the article.
